# IoT-Based Multi-Sensor Healthcare Architectures and a Lightweight-Based Privacy Scheme

**DOI:** 10.3390/s22114269

**Published:** 2022-06-03

**Authors:** Vassileios Aivaliotis, Kyriaki Tsantikidou, Nicolas Sklavos

**Affiliations:** SCYTALE Group, Computer Engineering and Informatics Department, University of Patras, 26504 Patras, Greece; vaivaliotis@ceid.upatras.gr (V.A.); k.tsantikidou@upatras.gr (K.T.)

**Keywords:** healthcare architectures, IoT, Health 4.0, lightweight privacy, cryptography, sensors

## Abstract

Health 4.0 is a new promising addition to the healthcare industry that innovatively includes the Internet of Things (IoT) and its heterogeneous devices and sensors. The result is the creation of numerous smart health applications that can be more effective, reliable, scalable and cost-efficient while facilitating people with their everyday life and health conditions. Nevertheless, without proper guidance, the employment of IoT-based health systems can be complicated, especially with regard to security challenges such susceptible application displays. An appropriate comprehension of the structure and the security demands of IoT-based multi-sensor systems and healthcare infrastructures must first be achieved. Furthermore, new architectures that provide lightweight, easily implementable and efficient approaches must be introduced. In this paper, an overview of IoT integration within the healthcare domain as well as a methodical analysis of efficient smart health frameworks, which mainly employ multiple resource and energy-constrained devices and sensors, will be presented. An additional concern of this paper will be the security requirements of these key IoT components and especially of their wireless communications. As a solution, a lightweight-based security scheme, which utilizes the lightweight cryptographic primitive LEAIoT, will be introduced. The proposed hardware-based design displays exceptional results compared to the original CPU-based implementation, with a 99.9% increase in key generation speed and 96.2% increase in encryption/decryption speed. Finally, because of its lightweight and flexible implementation and high-speed keys’ setup, it can compete with other common hardware-based cryptography architectures, where it achieves lower hardware utilization up to 87.9% with the lowest frequency and average throughput.

## 1. Introduction

In recent years, the demand for healthcare services that are high quality, low cost, widely available and easily accessible from the comfort of the patients’ homes is rapidly increasing. The result is the creation of notable problems that healthcare providers are struggling to resolve. These circumstances lead to the progressive inclusion of creative methodologies and technologies whose integration will greatly impact the healthcare industry. Some of these technologies are the Internet of Things (IoT), Internet of Services (IoS), Medical Cyber-Physical Systems, Cloud Computing, Fog, Big Data Analytics, 5G, Cryptography, Blockchain and Artificial Intelligence (AI).

Internet of Things (IoT) consists of heterogeneous devices that are interconnected with each other and with the Internet [1]. The utilization of IoT technology is rapidly growing with various systems emerging as efficient solutions to applicational restrictions [2]. It provides new services and intelligent capabilities by enabling the components to collect, store and analyze data from different resources while also retaining low-cost implementation. Specifically, the healthcare industry greatly benefits from these new services [3]. Health 4.0 introduces creative methods for the employment of innovative IoT technology [4]. Smart, cost-effective, reliable, scalable and easily accessible approaches, that facilitate multiple classes of people in various health-related situations, can be developed by following its main design principles, namely Interoperability, Virtualization, Decentralization, Real-Time Capability, Service Orientation and Modularity [5]. The first principle indicates the ability of the heterogeneous components to share data with each other and overall connect through Internet of Things (IoT) technology. Virtualization is another important principle that automates medical procedures by monitoring all healthcare elements and creating virtual copies of different processes. Decentralization requires all utilized healthcare devices to make decisions on their own and always execute their operations efficiently. Moreover, due to the critical nature of healthcare applications, the collection of data, decision making and healthcare functions must be executed in real-time without unnecessary delays, leading to the implementation of the Real-Time Capability principle. Service orientation implies that all operations and components of the Healthcare application are presented as services. These services can be accessible to all participants and they can be provided through various organizations. The last principle, Modularity, represents the flexibility of the system to constantly evolve and improve to fulfill new requirements and correspond to the scalability that characterizes IoT.

Nevertheless, the vision of Health 4.0, namely the integration of IoT technology and the healthcare industry, can add further difficulties to developers. The scalability and heterogeneous nature of IoT elements, the simultaneous utilization of numerous components and the delicate structure of healthcare systems can be a troublesome combination, especially without the proper comprehension of the architecture and operation of IoT-based healthcare infrastructures. Furthermore, security is an important factor in the development of IoT systems in healthcare [6]. The data that are collected by the sensors and are shared between the devices contain private information. Only authorized users must have access to the medical data, which must remain intact and efficiently encrypted throughout their transmission to the IoT smart health network. Their improper protection has legal and ethical implications because they can be exploited by attackers with the intention of possibly harming the user’s well-being. Moreover, only data from trusted devices and components must be accepted by the system in order to prevent the falsification of medical history and enhance the accuracy of remote diagnosis. The system must also be able to constantly execute its main functions without being obstructed by attacks, because even a small delay in critical operation can threaten the user’s life. For example, in smart health applications that require immediate responses for alerting medical services or providing first-aid assistance, specific types of attacks can delay these functionality processes and negate the system’s purpose of facilitating the patient and healthcare providers. Therefore, the application and maintenance of the main security schemes, such as confidentiality, availability and integrity, are a major concern for the research community.

The primary security challenges of IoT employment for smart health originate from various threats and vulnerabilities and are divided into two categories, namely embedded and network challenges [2]. The embedded challenges refer to the hardware and software aspects of IoT-based systems. The IoT components are resource- and energy-constrained devices that operate remotely and autonomously. By their own nature, they cannot support security algorithms that are resource and computationally demanding. Lightweight cryptography can resolve this problem because of the ability to adequately secure the architecture while utilizing fewer hardware resources. Nevertheless, in many cases, specific approaches that are used as a replacement are not as reliable as the well-known heavy cryptographic primitives [7]. Moreover, most smart health providers do not spend their resources achieving the security concepts, viz confidentiality, availability and integrity [8]. They rather only focus on the application’s healthcare functions and reduction in the implementational costs. Therefore, inept security practices and updates are adopted, resulting in an easily exploitable system. Another vulnerability originates from the scalable nature of IoT. Specifically, the devices are constantly being added to the system without the guaranty of their own integrity. An attacker can be granted access to the main system by compromising a small, unprotected device. Therefore, all devices, even the smallest ones, that interact with the central services must have a form of a lightweight security algorithm that protects them.

Network security challenges concern communication mechanisms and smooth data transmissions. All IoT devices continually connect and disconnect to a variety of communication networks that can either be private or unknown. In addition, the wireless networks utilized in IoT have many vulnerabilities on their own [9,10]. Their exploitation helps attackers intercept the network transmission and eavesdrop on the sensitive health data. Hence, they must implement flexible algorithms that are sufficiently capable to ensure the privacy and integrity of the collected data. This can be achieved by implementing encryption and decryption algorithms in the heterogeneous devices. However, the most common ciphers are resource-demanding and they cannot be utilized in the IoT environment. Furthermore, the scalability of IoT renders difficult the detection of malicious presences in the network, deeming necessary the employment of more complex data management schemes [11,12]. Despite the fact that the high production speed of the cipher key and quick encryption/decryption mechanism can alleviate some pressure on the transmission management, their achievement is challenging because of IoT hardware limitations. Lastly, a main challenge for IoT systems is the prevention and mitigation of Denial of Service (DoS) or Distributed Denial of Service (DDoS) attacks [2]. These attacks interfere with the communication processes and render the system unavailable to the end users by exploiting network vulnerabilities.

In recent years, many lightweight methods have been proposed for security, privacy and authenticity purposes in IoT-based healthcare applications. Some utilize new mechanisms such as Artificial Intelligence and Blockchain. However, these integrations add new vulnerabilities and implementational issues into the already security challenging IoT architecture [6]. Hence, they are not considered lightweight-efficient IoT-based security designs. Other implementations propose more flexible and appropriate approaches. The authors’ previous works proposed exemplary frameworks for limiting the implementation vulnerabilities and preventing possible attacks [13,14]. Other proposed architectures offered a variety of efficient security approaches that employ flexible security mechanisms and interactive cryptographic algorithms with minimum hardware resources [15,16].

In this paper, as a continuation of the authors’ research, an extensive study of healthcare frameworks and applications is presented. Afterwards, a basic architecture of an IoT-based multi-sensor healthcare infrastructure is demonstrated. The first contribution of this paper is this detailed analysis and calculated creation of an IoT-based healthcare structure which can benefit the research community by referencing this architecture and its properties in future related work. This general architecture has some security requirements and challenges regarding data transfers and encryption/decryption mechanisms. Specifically, the main security and functionality requirements are the following:

- A formal lightweight cryptographic primitive which can encrypt and decrypt the inputted messages with a considerable-sized key, thus ensuring the confidentiality, data privacy and authentication while deeming the decomposition of the algorithm through key search attacks to be difficult. The success of other known attacks will also be eliminated as each device will effectively secure the collected data before transmission.
- High speed in key generation and encryption/decryption processes for quick transmissions and the management of the significant IoT network load. Therefore, the constant availability of the architecture will be ensured because the communication system will be able to handle multiple messages that are rapidly transmitted.
- Flexible design with multiple options for key size and performance speed for various circumstances depending on the user preferences. The scalability of the IoT can thus be confronted by constantly adapting to the current needs of the system.
- Minimal resource utilization of the employed security scheme for efficient implementation in hardware-constrained IoT devices. This way, all devices in the healthcare structure can employ a level of security and hence ensure their integrity. Proper balance between the resources and the performance throughput must also be ensured. The performance flow of the security scheme must be considered when minimizing the resources of the design. The throughput must remain sufficiently high in order to correspond to high-speed transmissions.

A solution to these concerns is proposed and demonstrated. The proposed lightweight LEAIoT-based security scheme ensures the user authentication and the privacy of the collected data that are shared via IoT networks between resource-constrained devices and via the Internet to cloud-based services. Its resource-efficient implementation in IoT devices quickly encrypts the collected data with flexible and high-speed key generation options while adapting to various communication and computational needs. Therefore, the discussed requirements are fulfilled without adding pressure to the employed hardware-constrained components. The exact methods that lead to the fulfilment of the mentioned requirements in IoT-based healthcare architectures and thus the innovations and contributions of the proposed scheme are listed as follows:

- First, the employed cryptographic primitive, as presented in [17], ensures the maintenance of the main security concepts while being lightweight and having the ability to utilize various sized keys, from 32-bit to 256-bit length. The width of the key is a general parameter of the security level for symmetric encryption, deeming the 256-bit key highly effective for the proposed environment. The LEAIoT algorithm also utilizes asymmetric encryption, which further enhances the security and confidentiality of the system.
- Second, the high computation speed achieved by the hardware-based implementation ensures the quick network management of the IoT’s heavy communication load. The performance results of the proposed FPGA-based architecture are compared to the original LEAIoT design [17], which was implemented in a central processing unit (CPU), with a significant improvement of the key generation speed up to 99.9% and the encryption/decryption speed up to 96.2%.
- Third, in the same architecture, four different key sizes are integrated, providing four diverse performance speeds and security efficiencies that the applications can instantly choose from depending on the current network and the computational needs of the application. Therefore, the system offers flexibility and can easily adapt to various circumstances.
- Finally, the cryptographic scheme is properly implemented through innovative design optimizations and achieves novel implementational results that indicate its resource efficiency without an excessive reduction in throughput. Thus, the proposed scheme effectively fulfills the performance requirements for smart health and it can be easily employed to the various devices of the IoT network, providing global security to the healthcare structure while maintaining performance balance between resources, throughput and security. For further proving the architecture’s efficiency, the proposed hardware-based scheme is compared to other relative FPGA-based research. Specifically, it was concluded that the proposed implementation utilizes up to 87.9% and 76.9% fewer resources than the lightweight versions of Advance Encryption Standard (AES). It also decreases the resource consumption up to 65.7% and 12.2% compared to SNOW 3G and ZUC ciphers, respectively. Lastly, it achieves an almost double throughput compared to RC4 cipher and overall similar performance results with PRESENT and CLEFIA, even with a significantly lower frequency.

## 2. IoT-Based Multi-Sensor Healthcare Applications

### 2.1. Health 4.0 Objectives

**Figure 1:**
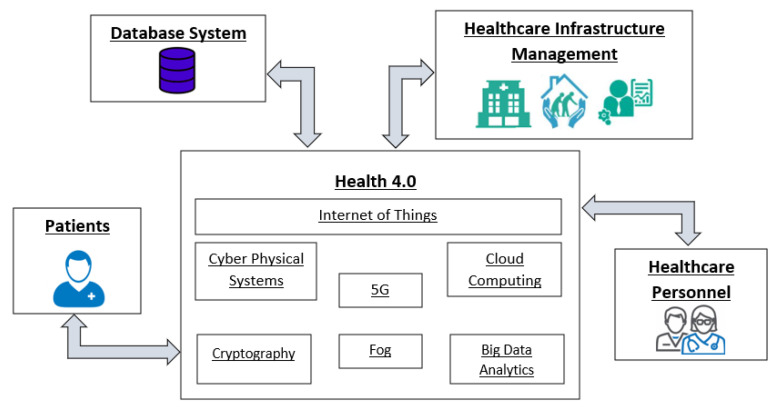
Health 4.0 framework.

Many smart health applications can be created by applying IoT technology, thus achieving the objectives of Health 4.0 [4]. A general framework for Health 4.0 depicting the integrated technologies and the main components of the healthcare architecture is presented in Figure 1. One of the most important objectives is the creation of high-quality services that facilitate people with various healthcare needs. This requires the improvement of the system performance, the efficient utilization of resources and the optimization of utilized tools. Automated procedures and intelligence can enhance the results’ accuracy and accelerate basic and repetitive tasks. Moreover, remote access and real-time responses can assist in immediate medical attention and constant monitoring. Finally, the design of appropriate databases with complete and easily accessible medical records can create a more personalized healthcare and a better diagnosis.

Another equally vital objective is the improvement of the operations’ effectiveness while simultaneously maintaining minimal costs as well as low resource utilization and energy consumption. Therefore, healthcare applications can be implemented in IoT devices that are mainly resource-constrained with energy limitations. However, the balance between the throughput and resource consumption is critical and depends on the requirements of the system. The ideal approach is the achievement of the highest performance throughput with the utilization of minimum resources. The employment of IoT, independently of its final optimizations, can assist in health monitoring, diagnosis and disease prediction through the enormous number of data that are collected by health sensors. These data are instantly transmitted, analyzed and saved to cloud services, making the diagnosis easier and more accurate. The pressure on healthcare personnel and materials will be reduced because the costly methods of health examination are replaced by cost-efficient, easily accessible, user-friendly and immediately responsive alternatives. Finally, the collaboration and sharing of information between multiple healthcare facilities and providers will be straightforward and timesaving.

### 2.2. IoT Architecture

**Figure 2:**
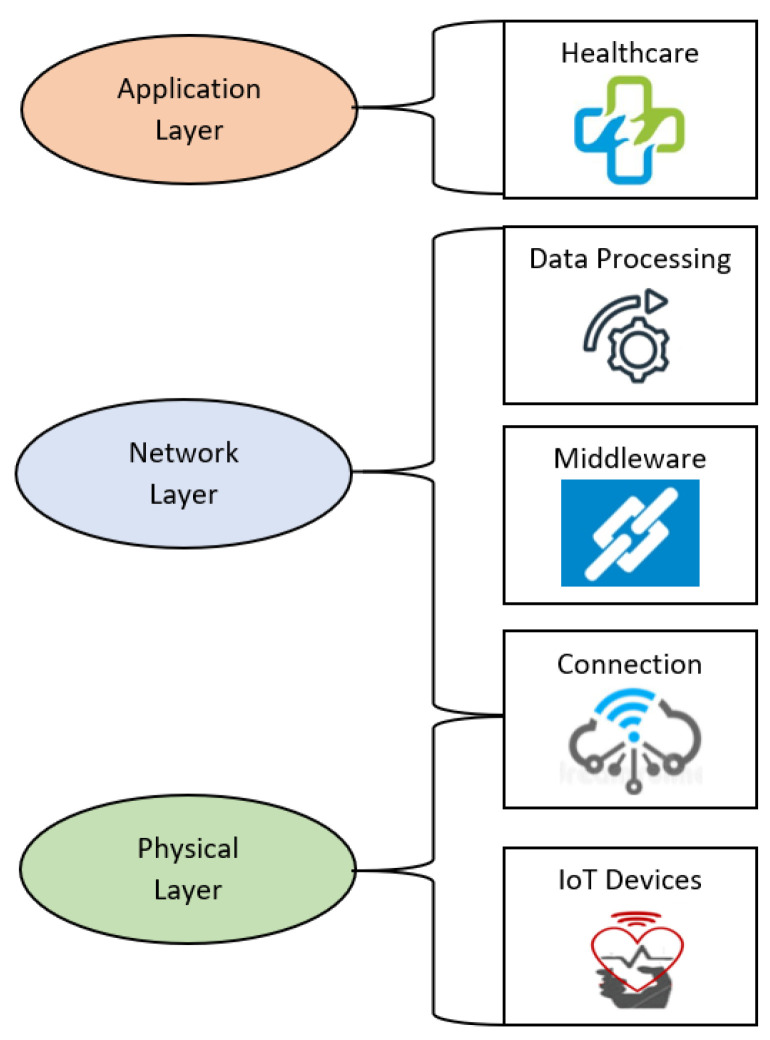
Architecture of IoT layers.

First, the IoT architecture is described by a three-layer design [11]. These three layers are the application layer, the network layer and the physical/perception layer. Figure 2 displays the IoT layers in a healthcare architecture with more details. The application layer is the highest layer in the architecture and connects the objects with the IoT network [11]. It consists of various healthcare applications that offer e-health services and functionalities to the user. The network layer enables the IoT components to connect with each other and share data which are collected by the devices of the physical layer through established communication protocols. Some commonly utilized networks are Bluetooth, ZigBee, 5G, Wi-Fi, Radio Frequency Identification (RFID), 6LoWPAN and LoRaWAN. Another established network of nodes that is integrated into the IoT is the Wireless Sensor Network (WSN) [18]. The physical/perception layer is the last layer of the architecture. It consists of all the physical objects employed in IoT systems such as sensors, wearables, actuators, smartphones, antennas, and processors, etc. The purpose of this layer is to collect health signals and convert them to readable data that the network layer can transmit.

### 2.3. Smart Health Multi-Sensors Designs from Literature

Healthcare services can now be provided outside the hospital facility with the employment of IoT technology. Remote health monitoring, telemedicine, the ambient-assisted living (ALL) of elderly or disabled people and supervision of chronic diseases are some crucial applications that can benefit healthcare [3,19,20]. Specifically, they can improve the effectiveness and accessibility of health services and help alleviate the pressure on hospital resources. Ref. [20] analyzed recent proposals for Internet of Health Things, ambient assisted living and remote healthcare monitoring systems. Furthermore, it provided illustrations of relative architectures. In [21], a decentralized architecture for a smart health system based on Internet of Medical Things was proposed. This design consists of three layers, namely the data producer layer, the hybrid computing layer and the data consumer layer. The first layer includes the IoT sensors producing health data which are later collected and transmitted over to a hybrid computing system benefitting from both edge and cloud paradigms. The decentralized data processing is achieved by utilizing Blockchain technology and Distributed Data Storage System (DDSS) methodology. Moreover, system privacy and security are established by introducing three cryptography algorithms. Another decentralized healthcare architecture was demonstrated in [22]. It aims to monetize the collected health data while offering guidelines for data security and privacy. The technologies employed, namely IoT, AI, Big Data and Blockchain, and all the architecture’s elements, were thoroughly described. Ref. [23] displayed the architecture of an IoT-based health application that manages Big Data. Each of its layers and key technologies were analyzed in depth. This design has been employed in smart clothing-based monitoring, telemedicine and emotion interaction services. In [24], a hardware-based implementation with multiple sensors connected to a main processor was designed. The system was employed for the constant monitoring of health variables.

The depiction and development of a Smart Hospital architecture is also a major concern for researchers. Ref. [25] proposed an architecture of a smart hospital that utilizes the low power wide area wireless protocol NarrowBand-IoT (NB-IoT). This protocol renders possible the communication between all IoT devices and sensors that are simultaneously employed in various hospital application scenarios. The paper thoroughly demonstrates the smart hospital’s infrastructure and applications. In [26], the implementation of an IoT-based multi-sensor monitoring system, which resembles previously mentioned remote architectures, is demonstrated. The design consists of three layers, namely the node layer that includes the Wireless Sensor Network (WSN) and sensors; the local management layer that is composed by the hospital’s computers; and the cloud-based layer, which collects and stores the data. A complete IoT-based tracking system applied in a hospital facility was implemented in [27]. The architecture was composed of multiple smart devices which recorded and transmitted the hand-washing data of the recognized subjects, smart ID cards for easy authentication of hospital personnel, hybrid routers and an IoT gateway for receiving and forwarding data to the last element, namely the cloud server, and sending commands in real-time. The purpose is the prevention of infections inside the hospital by tracking the hand-washing activities of hospital employees.

### 2.4. IoT-Based Multi-Sensor Healthcare Infrastructure

**Figure 3:**
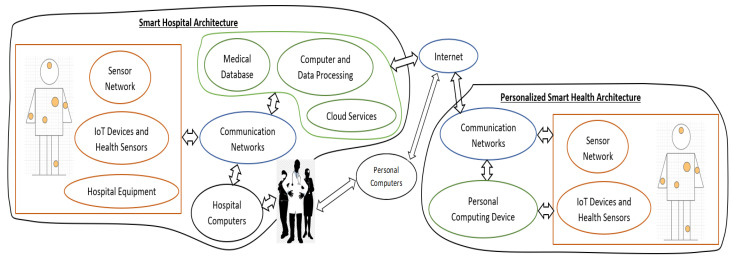
A general Smart Health architecture.

A complete IoT-based Health architecture is demonstrated in Figure 3. This general design depicts the relations between IoT components and healthcare elements. Smart hospitals, near-patient/personalized smart health systems as well as their connection are presented in detail. The personalized smart health architecture consists of multiple heterogeneous IoT devices, a wireless interface and a connection with a cloud-based database, as described in [2]. First, the devices that are utilized are mostly implantable or wearable medical devices and sensors, such as cardiac pacemakers or defibrillators, blood sugar/blood pressure/heart rate/electroencephalography (EEG) signal monitors, insulin pumps, etc. They are mostly battery dependent, making their implementation’s energy efficiency critical. Additionally, they must be able to connect to the wireless network of the system to transmit the “sensed” data. Numerous devices and sensors are simultaneously utilized and thus their interconnection is deemed necessary. Moreover, wearable devices must be portable, relatively small and comfortable while they are worn. Some of the implantable and wearable devices can be re-programmable through wireless communication or they can wirelessly receive various commands such as the adjustment of the drug dosage the device administrates or the configuration of the device settings. Furthermore, the wireless interface must connect to the Internet with the intention of transmitting the collected health data to healthcare personnel such as doctors and nurses. The processing of these data can be handled by the hospital’s or private clinic’s primary computing system, or by the near-patient IoT system. Some wearable devices may be able to process their collected data and afterwards wirelessly send them to the Internet. Alternatively, a more stationary or mobile device may be used to compensate for the small, wearable and implantable devices that do not possess processing capabilities. These intermediate devices first collect the data from multiple sensors which create the IoT system through the various established IoT networks, before completely or partially processing them and finally transmitting them to back-end systems and databases. Alternatively, they can receive authorized commands or data from back-end systems, process them and correspond through interaction with the sensors. Intelligence and real-time functionalities can be added by giving these devices the capability to make decisions and act accordingly without waiting for the back-end systems’ response. Moreover, all IoT devices have limited or no storage capacity. Thus, the data are transmitted and stored in databases to be easily accessible and create a complete medical history.

Nevertheless, IoT can also enhance hospital interactions and its inner functionality [25]. All the previously mentioned implantable and wearable IoT devices can also be utilized inside hospital facilities. Similarly, these multiple sensors and devices must be interconnected through wired and wireless networks and be able to receive from authorized elements or send to appropriate components commands and sensitive data. The main additional features of this hospital architecture are two. First, the clinical beds, which monitor the patient’s health through sensors, and all medical equipment, which collect the sensitive health data required for diagnosis, are given communication capabilities and added to the IoT network. Second, the IoT network is directly connected with the medical records systems and healthcare database located in the hospital facility. Hence, the hospital personnel can receive data in real-time and immediately respond to emergencies. Furthermore, the stored data can be easily accessed by all hospital equipment and promptly provide the patient’s medical history. Finally, the hospital’s IoT-based network must be connected to the Internet and cloud-based platforms in order to communicate with near-patient systems and other healthcare facilities. The result will be the creation of more personalized and intelligent health services that assist healthcare providers while reducing the stress on hospital resources.

## 3. Security Requirements

As previously discussed, security is an exceptionally important aspect in the implementation of IoT-based healthcare applications. However, IoT networks that are utilized in the general smart health infrastructure, Figure 3, can be subject to a variety of attacks, with the IoT device being its most susceptible component [2]. An attacker can easily gather the personal information shared by the devices via the IoT network. Eavesdropping and data transmission/traffic monitoring are two main attacks that breach data privacy [11]. Furthermore, without proper data protection, the user authentication is also disrupted. Unauthorized devices can gain access to these data that can then be processed or altered for malicious purposes [28]. They also can fabricate erroneous health data and transmit them to the other components of the IoT network. This leads to inaccurate health diagnosis and unreliable communication between the patient and health provider.

The development of secure communication networks is a major concern for researchers [29,30]. Cryptography is a commonly used practice for ensuring data privacy and user authentication [31]. It utilizes cryptographic algorithms, namely ciphers, for “hiding” the content of various texts through encryption and afterwards “revealing” the original data through decryption. Nevertheless, the known cryptographic primitives cannot be applied to the IoT system because of the devices’ resource constraints. The employed cipher must not deprive the valuable resources from other important functionalities that provide healthcare services. Therefore, a more lightweight version must be developed for properly corresponding to the hardware limitations of IoT. Furthermore, the delay of real-time applications because of the algorithms’ low speed implementation can be devastating in critical circumstances. Hence, the encryption/decryption speed and the systems’ immediate response must be taken into consideration. Finally, the system must provide multiple options with each functionality, satisfying different network and security needs depending on the application’s current requirements. Thus, flexibility and scalability mechanisms must be added to the system.

**Figure 4:**
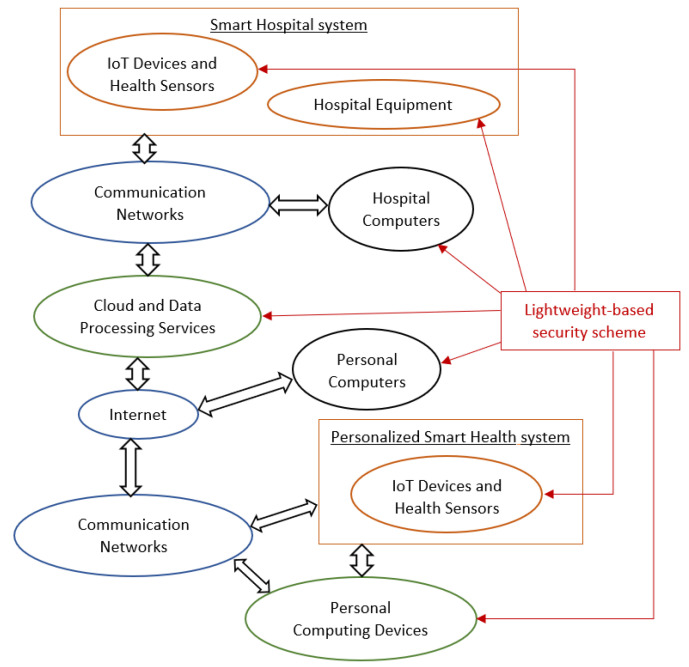
Lightweight-based security scheme.

Overall, for properly securing the health data of a smart health application, a lightweight cryptographic primitive must be implemented and a security scheme must be applied. First, the encryption algorithm must encrypt the collected data before they are shared between the utilized IoT devices. Thus, the patient’s personal information is protected from malicious attacks. Similarly, the decryption algorithm must decrypt these received data for their further employment depending on the healthcare application. The result is the total content protection of the transmitted data in the employed communication networks, specifically throughout the IoT networks and the Internet that is connected to cloud-based services. Figure 4 depicts all the possible components of a general smart health infrastructure that can implement a lightweight-based security scheme and fulfill all these mentioned requirements.

## 4. Lightweight-Based Security Scheme

The proposed lightweight-based security scheme employs the LEAIoT cryptographic primitive which efficiently encrypts and decrypts the collected data while providing flexible options regarding the key size and speed implementation. This mechanism is implemented into every IoT device that is utilized in a healthcare system, including both smart hospital and near-patient architectures, and provides data privacy to the communication networks and the Internet. Overall, the proposed lightweight-based security scheme can be applied as depicted in Figure 4.

### 4.1. LEAIoT: Lightweight Encryption/Decryption Algorithm

LEAIoT is a lightweight cryptographic primitive whose key generation and encryption/decryption speed are higher than other common encryption primitives [17]. The advantages of a lightweight design are fitting in the IoT-based healthcare environment, which has complex communication requirements. Specifically, it provides speed-efficient end-to-end communication with minimum hardware resource utilization. LEAIoT combines a symmetric encryption algorithm and an asymmetric encryption algorithm while aiming to preserve both of their advantages. The symmetric cryptography provides further operational speed with a minimum number of computing resources. The asymmetric primitive has better key distribution and scalability schemes and ensures the confidentiality and authentication of the system.

The LEAIoT encryption process of a plaintext—which is given a synthetic value—first employs the symmetric key encryption with a private key *n*. Both sender and receiver know the context of this key. Afterwards, the asymmetric linear block cipher (NLBC) is executed with the previously produced ciphertext and two keys, namely a shared key *n*_1_ and one private key *k*. The result is a completely secure encrypted text. The decryption sequence utilizes the modular inverse of the three encryption keys, via *SSK* (secure symmetric key), *n*_1_^(−1)^ and *k*′. First, the asymmetric—namely NLBC—decryption is performed with the transmitted ciphertext and the two keys, *n*_1_^(−1)^ and *k*′. These keys are produced by the modular inverse with modulo 37 of keys *n*_1_ and *k*, respectively. The original plaintext is generated by applying the symmetric decryption with the *SSK* key, which is the modular inverse of *n*. For this algorithm, the modular inverse operation is always executed with modulo 37. A detailed description of the encryption and decryption processes is followed.

Encryption sequence:

1. The synthetic values of the plaintext are multiplied with the private key *n*. Then, modulo 37 is used;
2. A 3 × 3 matrix is created as the private key *k*. Additionally, the length of the key *n* is calculated and utilized as the key *n*_1_;
3. The produced text from step 1 is segregated into blocks *b_i_* matching the key *k*. Specifically, it is divided to multiple arrays of length equal to 3;
4. Each block *b_i_* is multiplied with the keys *k* and *n*_1_. Modulo 37 is subsequently applied;
5. The result is the secure ciphertext of the original plaintext;

Decryption sequence:

1. The modular inverse of the keys *n_1_* and *k* with modulo 37 is utilized;
2. The received ciphertext is divided to blocks *b_i_*, as with step 3 of the encryption sequence;
3. Each block is multiplied with the two keys *k*′ and *n*_1_^(−1)^. Modulo 37 is then applied;
4. The produced text is afterwards multiplied with the key *SSK* and modulo 37 is once again used;
5. The result is the original plaintext.

A simplified example of the encryption and decryption calculation processes is presented for a better comprehension of these two procedures. The private key *n* is 12,345, the inputted synthetic values are $\left[\begin{array}{c} 13\\ 19\\ 15 \end{array}\right]$, and the private key *k* is $\left[\begin{array}{ccc} 1 & 2 & 1\\ 4 & 5 & 6\\ 7 & 8 & 9 \end{array}\right]$.

Encryption sequence:

1. First, the inputted values are multiplied with key *n*:
  $$\left(\left[\begin{array}{c} 13\\ 19\\ 15 \end{array}\right]*12345\right)\;mod\;37=\left[\begin{array}{c} 16\\ 12\\ 27 \end{array}\right]$$
2. Finally, the result of the previous calculation is multiplied with key *k* and the length of key *n* to produce the encrypted message:
  $$\left(\left[\begin{array}{ccc} 1 & 2 & 1\\ 4 & 5 & 6\\ 7 & 8 & 9 \end{array}\right]*\left[\begin{array}{c} 16\\ 12\\ 27 \end{array}\right]*5\right)\;mod\;37=\left[\begin{array}{c} 2\\ 24\\ 35 \end{array}\right]$$

Decryption sequence:

1. First, the *SSK*, *k*′ and *n*_1_^(−1)^ are calculated:
  $$SSK=\left(n^{\left(-1\right)}\right)\;mod\;37=12345^{\left(-1\right)}\;mod\;37=17\;n_{1}{}^{\left(-1\right)}=\left(5^{\left(-1\right)}\right)\;mod\;37=15\;k^{\prime }=k^{\left(-1\right)}=\left[\begin{array}{ccc} 18 & 23 & 32\\ 1 & 25 & 12\\ 18 & 1 & 18 \end{array}\right]$$
2. Then, the encrypted message is multiplied with *k*′ and *n*_1_^(−1)^:
  $$\left(\left[\begin{array}{ccc} 18 & 23 & 32\\ 1 & 25 & 12\\ 18 & 1 & 18 \end{array}\right]*\left[\begin{array}{c} 2\\ 24\\ 35 \end{array}\right]*15\right)\;mod\;37=\left[\begin{array}{c} 16\\ 12\\ 27 \end{array}\right]$$
3. Finally, the original message is revealed by multiplying the previous result with the *SSK* key:
  $$\left(\left[\begin{array}{c} 16\\ 12\\ 27 \end{array}\right]*17\right)\;mod\;37=\left[\begin{array}{c} 13\\ 19\\ 15 \end{array}\right]$$

### 4.2. Implementation of the Proposed Lightweight-Based Security Scheme

**Figure 5:**
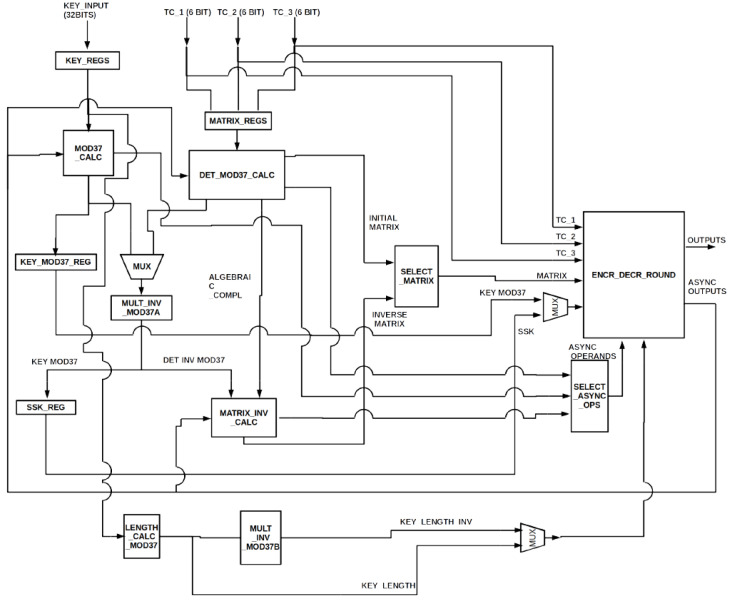
Proposed architecture of cryptographic primitive.

In this section, the proposed lightweight-based security scheme is presented. The LEAIoT-based lightweight encryption/decryption implementation has a symmetric and asymmetric encryption and decryption stages. The user can choose the length of the symmetric key between 32, 64, 128 or 256 bits. For the NLBC or asymmetric phase, the size of the private key *k* is a 3 × 3 matrix with each element having a value within the range of [1, 36]. The overall design of the proposed cryptographic approach is demonstrated in Figure 5. The architecture has additional input and output signals that are not depicted in this figure. The extra input signals concern the start of a new procedure, the selection of the procedure that will be executed and the selection of the key size. The supplementary output signals indicate the completion of each key generation procedure, the encryption process and the decryption process.

The main units of the architecture are analyzed below.

- ENCR_DECR_ROUND unit contains the necessary resources for the encryption and decryption of the input text. It also executes the additions and multiplications of finite elements and sends the results into the appropriate units.
- The MOD37_CALC unit calculates modulo 37 of symmetric key *n*.
- DET_MOD37_CALC unit’s input is a 3 × 3 matrix, namely key *k*, and its outputs are modulo 37 of the matrix’s determinant and table I, which is an intermediate step for computing the modular inverse of the matrix. Each element of table I, namely *i_ab_*, is calculated by the following equation:
  (1) $$\;\;i_{ab}=det\;2_{ab}*\left({\left(-1\right)}^{\left(a+b\right)}\right)\;mod\;37,$$
  where $det\;2_{ab}$ is the determinant of a 2 × 2 matrix created after erasing row a and column b of the input matrix.

- MULT_INV_MOD37 unit computes the modular inverse of the input. The input can either be module 37 of the symmetric key *n* or the determinant’s module 37 of key *k*, which was calculated by DET_MOD37_CALC. The input is selected by a multiplexer.
- MATRIX_INV_CALC unit receives the determinant’s modular inverse of key *k* as input, as computed by MULT_INV_MOD37, and receives the intermediate table I as input, as calculated by DET_MOD37_CALC. The output is the modular inverse of key *k*.
- LENGTH_CALC_MOD37 unit calculates the length of symmetric key *n*, namely generating key *n*_1_. The modular inverse of this key can be produced by the MULT_INV_MOD37 unit. The procedure is the same as with the modular inverse computation of symmetric key *n*.

The four main functions of the proposed lightweight architecture are the insertion of a new symmetric key *n*; insertion of a new asymmetric key *k*; encryption; and decryption. The first function changes the key *n* of the symmetric encryption/decryption stage and calculates the new *SSK*. The user selects the length of the new key, namely 32, 64, 128 or 256 bits. The key is inserted in blocks of 32-bit and is stored in the registers KEY_REGS. The contents of registers KEY_REGS are then given as inputs to the MOD37_CALC, which calculates the n mod 37. The result is stored in the register KEY_MOD37_REGS, which is the input of the MULT_INV_MOD37A unit. This unit calculates the $n^{-1}\;mod\;37$, namely the *SSK* key. The produced *SSK* key is stored in the register SSK_REG. Finally, the units LENGTH_CALC_MOD37 and MULT_INV_MOD37B calculate the $n_{1}\;mod\;37$ and $n_{1}^{\left(-1\right)}\;mod\;37$, respectively.

The second function, namely the insertion of a new asymmetric key *k*, changes the key *k* and calculates its modular inverse, namely *k′*. The insertion of the key, which is a 3 × 3 matrix, is executed in three stages. In each stage, the three elements of a row are inserted via the three inputs, tc_1, tc_2 and tc_3. The elements are stored in the registers MATRIX_REGS. The completely inserted key is then given as input in the DET_MOD37_CALC unit. This unit produces two outputs, namely the determinant’s module 37 of the key and table I. The first output is inserted to the MULT_INV_MOD37A unit, which calculates its modular inverse. The second output is given as one of the two inputs of the MATRIX_INV_CALC unit. The other input is produced by the MULT_INV_MOD37A unit modular inverse of the determinant. The final output is the modular inverse of the new asymmetric key *k*, namely *k′*.

The encryption function begins by inserting three characters of the plaintext via the inputs tc_1, tc_2 and tc_3 to the ENCR_DECR_ROUND unit. The other inputs of this unit are modulo 37 of key *n*; modulo 37 of the *n* key’s length; and key *k*. The result is the ciphertext which is computed by the correct processing of the data. The final function, namely decryption, starts by inserting three characters of the ciphertext via the three inputs tc_1, tc_2 and tc_3 to the ENCR_DECR_ROUND. The other inputs of this unit are the modular inverse of the key *n* (*SSK*), the modular inverse of the key *n* length (*n_1_*^(−1)^) and the modular inverse of the key *k* (*k′*).

#### 4.2.1. Modulo 37 Calculation Module

The basic element of this module is an asynchronous unit that calculates module 37 of a 32-bit number. Its operation is based on the algorithm for modulo reduction that was proposed in [32]. Moreover, the multiplications are implemented based on the Wallace method for multiplying integers using only combinational logic [33]. There are three steps for the module’s process. First, the key is divided into 32-bit sections and modulo 37 of each section is calculated. The results are stored in registers. For 32-bit keys, the process is completed with this step. The calculation and data storage of each section is completed in one clock cycle.

Afterwards, modulo 37 of each section—excluding the 32 least important bits—is multiplied with modulo 37 of a power of 2, which depends on its position. Specifically, for module 37 of the [*n*, *n* + 31] bits, the operation $2^{n}\;mod\;37$ is used. Then, the results are stored in registers. For utilized hardware reduction purposes, the multiplications of module 37 are only implemented in the encryption/decryption unit and not in this unit. The encryption/decryption unit can execute three modulo 37 multiplications anytime. Hence, the process is completed after a clock cycle for 64-bit or 128-bit keys, because one and three multiplications are needed, respectively. For a 256-bit key, seven multiplications are required, thus the process is completed after three clock cycles.

Finally, the sum of the previous results’ modulo 37 is calculated. Similar with the multiplications, the additions are only implemented in the encryption/decryption unit, with three of them being ready anytime. For a 64-bit key, only one addition is executed and its completion requires one clock cycle. For a 128-bit key, two clock cycles are needed. Finally, for the 256-bit key, four clock cycles are required.

#### 4.2.2. Three-Dimensional Matrix’s Determinant Calculation Module

The input of this module is a 3 × 3 matrix M and its outputs are the determinant’s module 37 and table I. The calculation process also follows three steps. First, the data of the input matrix M are inserted into the module that produces table I. After the computation of element *I*_11_, this same element and *M*_11_ are sent to the encryption/decryption unit. The unit calculates modulo 37 of their multiplication and stores the result in a register. In a similar way, the operations $M_{12}*D_{12}\;mod\;37$ and $M_{13}*I_{13}\;mod\;37$ are computed and stored. It is important to clarify that the two elements *I*_12_ and *D*_12_ are generated simultaneously. After the complete computation of table I, the results from the two operations $M_{11}*I_{11}\;mod\;37$ and $M_{13}*I_{13}\;mod\;37$ are sent to the encryption/decryption unit. Modulo 37 of their sum (*S*) is calculated. Finally, the difference $Diff=S-\left(M_{12}*D_{12}\;mod\;37\right)$ is computed and the process is completed.

The module that produces table I executes the following procedure. First, the matrix M elements are stored in registers. Afterwards, the cells of table I are generated sequentially. The $i_{ab}$ elements are produced by the det2 × 2 module. This module receives the data of the 2 × 2 matrix, which is generated from the input matrix M and calculates modulo 37 of the input’s determinant. If ${\left(-1\right)}^{\left(a+b\right)}=1$, then the requested element of table I is the output of det2 × 2, which is stored in a register. If ${\left(-1\right)}^{\left(a+b\right)}=-1$, then the output of det2 × 2 is subtracted from the value 37. This difference is the requested element. It must be clarified that, during the computation of $i_{12}$, both the output of the det2 × 2 module, which is utilized in the calculation of the M matrix’s determinant, and the result of this output subtracting with the value 37, which is the element $i_{12}$, are stored in registers.

The two-dimensional matrix’s determinant calculation module receives a 2 × 2 E matrix as input and generates modulo 37 of the input’s determinant. First, the E matrix’s elements are stored in registers. These elements are sent to the encryption/decryption unit, which calculates and stores the values $p_{1}=E_{11}*E_{22}\;mod\;37$ and $p_{2}=E_{12}*E_{21}\;mod\;37$ in registers. Afterwards, the value $d_{1}=p_{1}-p_{2}$ is computed. If $d_{1}\geq 0$, then the E matrix’s determinant is equal the value $d_{1}$, which is stored to the output register, and the calculation is completed. If $d_{1}<0$, then value $d_{2}=p_{2}-p_{1}$ is calculated and stored in the output register. Lastly, the value $d^{\prime }=37-d_{2}$ is computed and stored in the output register, thus completing the operation.

#### 4.2.3. Modular Inverse Calculation Module and Three-Dimensional Matrix’s Modular Inverse Calculation Module

The modular inverse calculation module, which exclusively uses combinational logic, accepts as input a 6-bit value (*a*) in the range of [0, 36] and returns the value *b* where $\left(a*b\right)\;mod\;37=1$. A system of logic gates assists the module in identifying the value and then selecting the appropriate output *b* with the usage of multiplexers.

The three-dimensional matrix’s modular inverse calculation module receives as input table I and the modular inverse of the M matrix’s determinant. The output is the modular inverse of the M matrix (M’). The M’ matrix’s elements of each row are calculated by multiplying the elements in table I’s corresponding column with the modular inverse of the determinant. Furthermore, the multiplications are executed in the encryption/decryption unit. Therefore, the process is completed after three clock cycles since three multiplications can be executed anytime table I is a 3 × 3 matrix.

#### 4.2.4. Encryption and Decryption Unit

**Figure 6:**
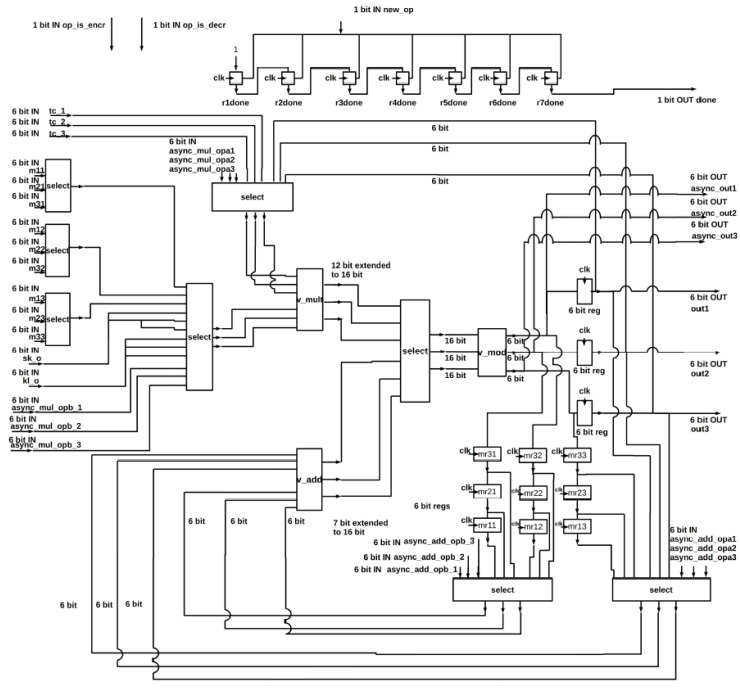
The architecture of the encryption and decryption unit.

The proposed architecture of the encryption and decryption unit is depicted in Figure 6. The inputs are the following:

- A 3 × 3 matrix with 6-bit elements (m11, m12, …, m33);
- Three 6-bit characters (tc_1, tc_2, tc_3) from the text which will be encrypted or decrypted;
- A 6-bit key (sk_o);

- A 6-bit signal (kl_o), which is modulo 37 of the key length or its modular inverse;
- Three single bit signals that indicate the start of a new operation or the applied process, namely encryption or decryption;
- Twelve different data for the generation of the keys, six of them (async_add_opa1, async_add_opa2, async_add_opa3, async_add_opb1, async_add_opb2, async_add_opb3) are added together while the other six (async_mul_opa1, async_mul_opa2, async_mul_opa3, async_mul_opb1, async_mul_opb2, async_mul_opb3) are multiplied with each other;
- A signal (async_op_is_add) which selects whether the operation for the keys’ generation is addition or multiplication.

Moreover, the basic modules of this unit are the v_add, v_mul and v_mod. The first one consists of three parallel additions. The second one consists of three parallel multiplications, which are implemented based on the Wallace algorithm. The last one consists of three smaller versions of the modulo computation, which is based on the Barrett algorithm.

In the first clock cycle of the encryption process, the inputs tc_1, tc_2 and tc_3 are multiplied with the sk_o in the v_mult module. The results are then sent to the v_mod module, which calculates modulo 37. The v_mod module’s outputs are stored in the output registers. For the next three cycles, each row of the input matrix is multiplied with the array that contains the values of the output registers element by element. Modulo 37 of the produced values is computed and then stored in the registers mr11, …, mr33. In the next two cycles, the columns of the matrix, which contain the values of registers mr11, …, mr33, are being added together in the v_add module, their modulo is computed in v_mod and the result is stored in the output registers. Finally, the values of the output registers are multiplied with the kl_o, their modulo 37 is calculated, the results are stored in the output registers afresh and the signal, which indicates the process completion, is generated.

For the first three clock cycles of the decryption process, each row of the input matrix is multiplied element by element with the array [tc_1, tc_2, tc_3], modulo 37 of the produced values is computed, and the results are stored in register mr11, …, mr33. In the next two cycles, the columns of the matrix, which contain the values of registers mr11, …, mr33, are being added together, their modulo 37 is calculated and the results are stored in the output registers. The values of the output registers are multiplied with the kl_o and modulo 37 of the produced result is stored in the output registers. Finally, the new values of the output registers are multiplied with the ks_o, modulo 37 of the results is stored in the output registers afresh and the signal indicating the process completion is generated.

Additions and multiplications with modulo 37 are required for both the generation of the keys and the encryption and decryption procedure. For efficient hardware utilization, the modules of the encryption/decryption unit are also employed for the key generation functions. Therefore, the unit executes the appropriate additions and multiplications with the twelve given inputs (async_add_opa1, async_add_opa2, async_add_opa3, async_add_opb1, async_add_opb2, async_add_opb3, async_mul_opa1, async_mul_opa2, async_mul_opa3, async_mul_opb1, async_mul_opb2, async_mul_opb3) and returns the results to the key generation modules via the outputs async_out1, async_out2 and async_out3.

## 5. Synthesis Implementation Results

The proposed architecture is simulated and implemented in FPGA 7z007s-clg400 [34] using VHDL. This FPGA was selected because it had the fewest hardware resources in the Vivado web pack 2018.3 [35]. For the simulation, the ghdl tool [36] was utilized, and for the synthesis and implementation, the Vivado web pack 2018.3 tool was used.

### 5.1. Simulations

**Figure 7:**
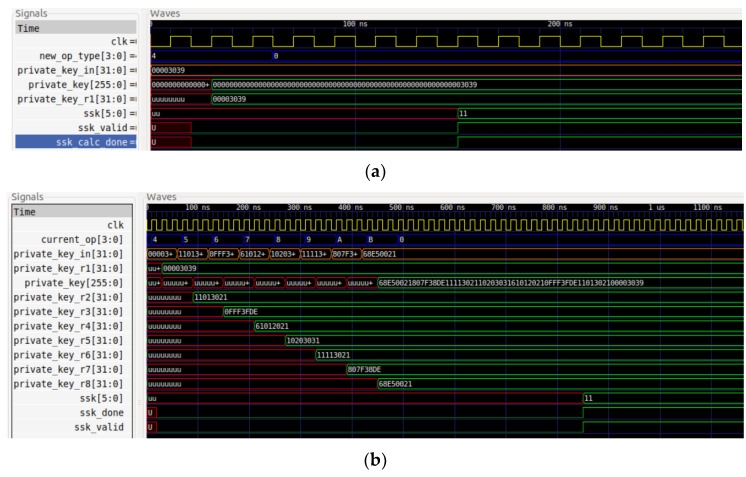
Insertion and modular inverse calculation of the symmetric key with: (**a**) 32-bit; and (**b**) 256-bit size.

**Figure 8:**
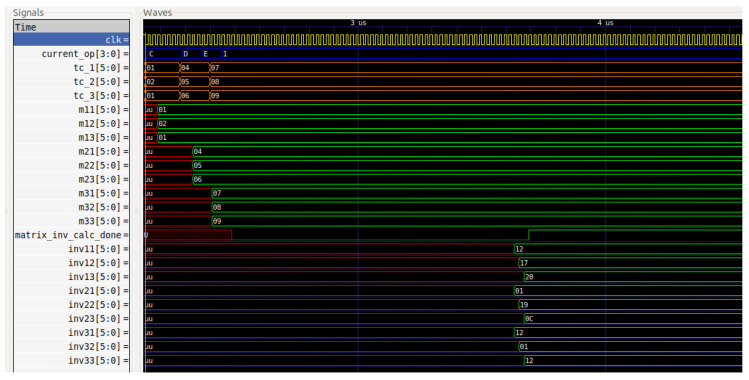
The modular inverse calculation of the asymmetric key.

The insertion and the modular inverse calculation of the symmetric key and the asymmetric key are simulated. For a better comprehension of the two operations, Figure 7 and Figure 8 present some examples for various key sizes.

The operations for inserting the new symmetric keys are denoted by the values 4_hex_ for a 32-bit key and [4_hex_, B_hex_] for a 256-bit key. The complete insertion of the matrix, namely the asymmetric key, is denoted by the values C_hex_, D_hex_ and E_hex_. Moreover, the 0_hex_ and 1_hex_ indicate the start of the modular inverse operation.

**Figure 9:**
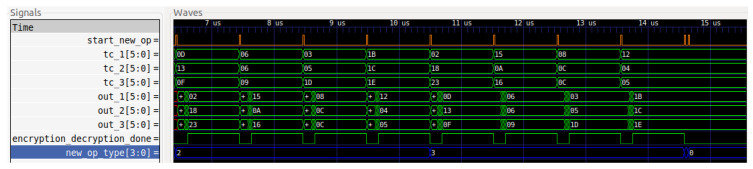
Encryption and decryption operations.

The encryption, which is denoted with the 2_hex_ value, and the decryption, which is denoted with the 3_hex_ value, are also simulated, as demonstrated in Figure 9.

### 5.2. Implementation

The proposed architecture of the encryption and decryption processes three 6-bit characters in each iteration. Furthermore, each function requires a total of nine clock cycles. The first cycle is needed to properly start the encryption or decryption operation and in the next eight cycles, the operation is executed. The results of the proposed implementations are presented in Table 1. The clock cycles required for the generation of both keys are displayed in Table 2. In these clock cycles, the cycles needed for the start signal generation are included.

## 6. Performance Results

The proposed architecture of the LEAIoT-based encryption and decryption process is resource and speed efficient. Therefore, it can be employed in IoT-based healthcare applications for lightweight security in the communication network. Moreover, the generation speed of the symmetric and asymmetric keys is exceptionally high. The implementational results, as presented in Table 3, indicate the architecture’s superiority over the original CPU-based design in [17]. Specifically, the proposed design has 99.9% higher key generation speed than the original for all key sizes and it achieves 99.3%, 98.4%, 97.5% and 96.2% higher encryption/decryption speed for 100 kilobits, 300 kilobits, 500 kilobits and 1000 kilobits, respectively. There are two reasons for these great differences in key generation speed. The first is the speed acceleration ability of FPGAs over CPUs. A CPU is a chip that executes the instructions of a program sequentially, whereas an FPGA is a configurable chip consisting of logic blocks which is ideal for the parallelization and simultaneous execution of instructions. Thus, the FPGA can be properly modified to execute the specific operations of the program better and faster in a parallel manner in contrast to the general-purpose CPU. The last reason is the design efficiency of the proposed methodology which parallelizes the sequential calculations of the algorithm and exploits the optimized nature of the FPGA.

To the best of the authors’ knowledge, there are no other implementations of the lightweight encryption algorithm LEAIoT. Thus, this architecture is the most lightweight and time-efficient implementation of LEAIoT. It also has four different key selection choices that provide flexibility in the system. Hence, depending on the application’s requirements, the latency and security of the architecture can be configured.

Furthermore, the proposed architecture is compared to various cryptography implementations from the literature, as presented in Table 4. The comparison parameters are the key size which can be a general representative of the provided security level, the number of implemented Look-Up Tables (LUTs), which are custom truth tables that determine the output value for given inputs and can be compared to a small RAM, and the number of utilized Flip-Flops (FFs), which are binary shift registers that store logical states between the system’s clock cycles. Moreover, the frequency and throughput of all these designs, which represent the system’s performance efficiency, are also compared for better examining the proposed architecture’s execution and implementation advantages.

All the compared cryptography designs are developed in different FPGAs with higher frequencies, contrasting the LEAIoT implementation. Therefore, a proper comparison of the architectures cannot be achieved. Nevertheless, for further proving the efficiency of the proposed system, a typical comparison is performed. First, even though the proposed design has the lowest frequency, it achieves better hardware consumption than most of the other ciphers. It utilizes 87.9% and 76.9% fewer resources than the lightweight versions of AES. It also achieves a 65.7% and 12.2% decrease in resource consumption compared to SNOW 3G and ZUC ciphers. Moreover, it achieves an almost double throughput than RC4 cipher, even with its comparatively lower frequency. Lastly, the proposed architecture has relatively similar performance results with PRESENT and CLEFIA. One significant difference is the lower frequency of the LEAIoT design. Thus, it can be concluded that the hardware and throughput efficiency of the proposed implementation can surpass the PRESENT and CLEFIA approaches by simply increasing the frequency.

Finally, the proposed design will be evaluated depending on the security and performance requirements that were previously clarified. The employed cryptographic primitive was tested and the provided security was verified through simulation. It has also been implemented with four different key sizes that the user can choose from in order to provide configuration abilities and flexibility for constantly corresponding to varying network speeds and security needs. For example, when the network is overloaded, a smaller key size can be selected to accelerate the encryption process even more. Likewise, for crucially important messages, a higher key size can be utilized to efficiently protect the contents of the data. Furthermore, the key generation and encryption/decryption speed have been accelerated offering great transmission speed to the system and facilitating the maintenance of the system’s constant availability. Hence, it can respond to the high-speed demands of the IoT network. Lastly, the synthesis’s results together with the comparisons with other related hardware-based works proved the lightweight capability and performance efficiency of the proposed architecture required for IoT-based healthcare systems. The design achieves an effective balance between resources, throughput and security, presenting a novel security implementation for the analyzed IoT-based healthcare architecture.

## 7. Conclusions

In this paper, the IoT-based multi-sensor architecture was analyzed. This was first achieved by demonstrating the Health 4.0 design framework and investigating the smart health infrastructure. This extensive presentation of the particular environment offers a proper guidance to efficiently employing IoT in the healthcare domain, either for smart hospitals or for personalized smart health systems. Furthermore, a deep comprehension of the domain’s current state was achieved with the display of representative research. Security, as one of the main concerns in IoT, was also discussed. The maintenance of data privacy and user authentication is the most important requirement of general smart health infrastructure. This has led to the proposition of a new hardware-based security implementation suitable for IoT devices. The proposed lightweight-based security scheme, which utilizes the LEAIoT encryption/decryption algorithm, provides more key selection possibilities than other compared architectures. Hence, it can offer extra speed when the network is overloaded. Furthermore, its hardware-based design has 99.9% higher key generation speed and 96.2% higher encryption/decryption speed for 1000 kilobits compared to the original CPU-based LEAIoT implementation presented in [17]. Moreover, it is resource-efficient because it employs the fewest number of FFs and a comparative small number of LUTs. Specifically, it achieves a 87.9%, 65.7% and 12.2% decrease in hardware resources compared to the lightweight ciphers, AES, SNOW 3G and ZUC, respectively, which were implemented in similar hardware devices. This trait can be efficiently utilized in an IoT-based multi-sensor environment because even the smallest devices can execute this design and be secured. Lastly, even though it has low throughput, it also has one of the lowest frequencies which better represents the resource limitations of IoT devices. Overall, it is best suited for applications that prioritize resource-efficiency and the high-speed generation of multiple keys. It has also proven capable of efficiently satisfying the main security and performance requirements of the introduced IoT-based smart health structure, providing innovative results and optimizations.

Future works will aim at both design improvements and further application employment. Specifically, the increase in the frequency and the matrix’s size are the next two steps that will enhance the efficiency of the implementation. Finally, another future objective will be the simulation of this security architecture to IoT-based environments that collect and transmit healthcare data in real time.

## Figures and Tables

**Table 1:** The characteristics of the proposed lightweight-based security scheme.

LUTs	Flip-Flops	F7-Muxes	F8-Muxes	Execution Time	Frequency	Throughput	Throughput/Slices
2700	815	67	24	261 ns	34.483 MHz	68.965 Mbps	0.0191

**Table 2:** Clock cycles for the generation of keys.

S.K. * Size (bit)	Insertion of S.K. *	Modular Inverse of S.K. *	Insertion of A.K. **	Modular Inverse of A.K. **	Total
32	2	5	6	61	74
64	4	8	6	61	79
128	8	12	6	61	87
256	16	19	6	61	102

* symmetric key. ** asymmetric key.

**Table 3:** Performance comparisons with the original design in [17].

Operation	Original CPU-Based LEAIoT [17] (ms)	The Proposed FPGA-Based Architecture (ms)
32-bit key generation	4	0.002146
64-bit key generation	10	0.002291
128-bit key generation	16	0.002523
256-bit key generation	22	0.002958
Encr/Decr * of 100 kilobits	210	1.45
Encr/Decr * of 300 kilobits	275	4.35
Encr/Decr * of 500 kilobits	290	7.25
Encr/Decr * of 1000 kilobits	386	14.5

* encryption/decryption.

**Table 4:** Performance comparisons from the literature.

Algorithm	Key Size	LUTs	FFs	Frequency (MHz)	Throughput (Mbps)
SNOW 3G [37]	128	7881	2391	28.84	922.88
AES-128 [38]	128	20402/14798	8704/1345	332.34/272.33	4342/3485
AES-MPPRM [39]	128	8129	7119	81.328	42.92
PRESENT [40]	80/128	215/264	153/201	213.81/194.63	102.89/91.59
ZUC [41]	256	2494	1512	209.346	6540
CLEFIA [42]	128/192/256	1725	663	147	990/818/696
RC4 [43]	-	277	197	123.64	41.21
Proposed design	32/64/128/256	2700	815	34.483	68.965

## Data Availability

All of the reported data are included in the manuscript.
